# From ophthalmologic evaluation to the diagnosis of ectopic adrenocorticotropic hormone syndrome

**DOI:** 10.1210/jcemcr/luag128

**Published:** 2026-05-29

**Authors:** Adnan Haider, Omar Sadat, Nicole Pumariega, Kevin Eid

**Affiliations:** Division of Endocrinology and Metabolism, Department of Medicine, West Virginia University School of Medicine, Morgantown, WV 26505, USA; Department of Ophthalmology and Visual Sciences, West Virginia University School of Medicine, Morgantown, WV 26505, USA; Department of Ophthalmology and Visual Sciences, West Virginia University School of Medicine, Morgantown, WV 26505, USA; Department of Ophthalmology, John H. Stroger Jr. Hospital of Cook County, Chicago, IL 60612, USA

**Keywords:** endogenous Cushing syndrome, central serous chorioretinopathy

## Image legend

**Figure 1:**
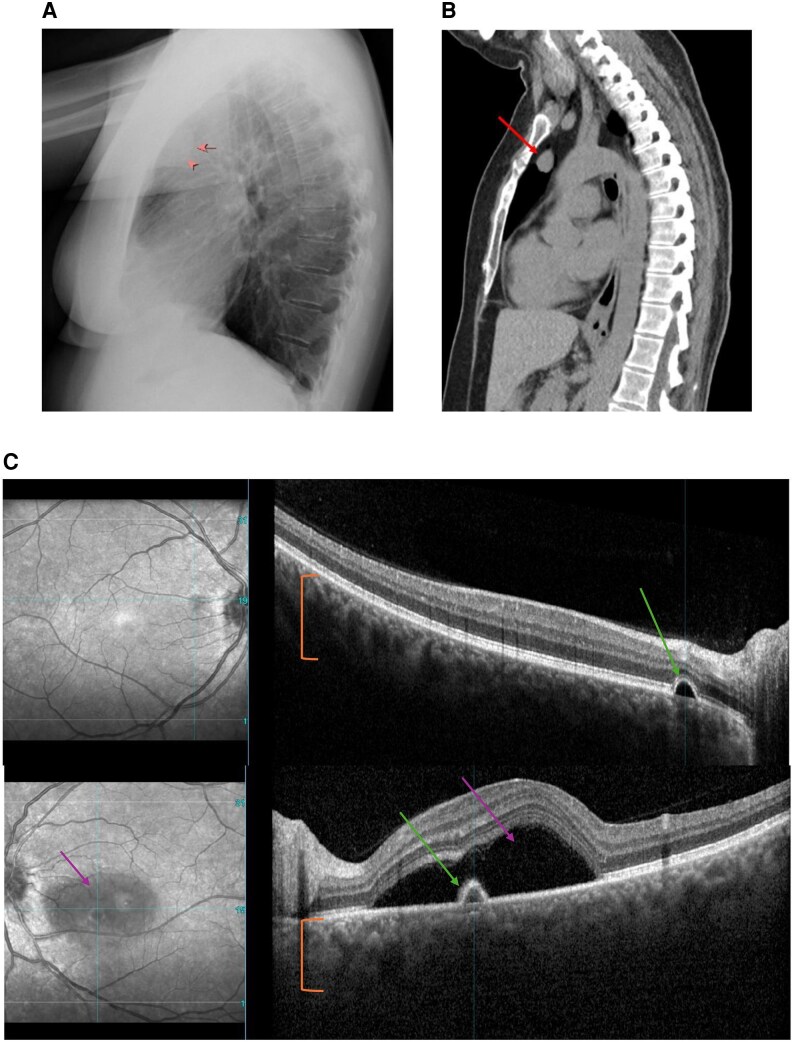
(A, B) Chest imaging demonstrating a mediastinal mass (red arrows). (C) Optical coherence tomography of the left eye demonstrating findings consistent with central serous chorioretinopathy, including pigment epithelial detachments (green arrows), subretinal fluid (purple arrows), and choroidal thickening (orange bracket).

A 44-year-old woman presented with intermittent left eye blurriness and brown spots in her visual field. Four weeks earlier, she was evaluated for facial swelling and ankle edema, and she was noted to be hypertensive. Chest x-ray and computed tomography identified a mediastinal mass (Fig. 1A and 1B, arrows). Ophthalmologic evaluation demonstrated central serous chorioretinopathy (CSCR) (Fig. 1C). Green arrows indicate pigment epithelial detachments, purple arrows indicate subretinal fluid, and the orange bracket highlights choroidal thickening. Biochemical evaluation showed a 24-hour urine free cortisol was elevated at 2988 µg/24-h (SI: 1083 nmol/24-h) (reference range, 4-50 µg/24-h [SI: 11-138 nmol/24-h]). Morning cortisol after a 2 mg dexamethasone was 32.6 µg/dL (SI: 899 nmol/L), and adrenocorticotropic hormone (ACTH) was elevated at 193.6 pg/mL (SI: 42.5 pmol/L) (reference range, 6.6-65.0 pg/mL [SI: 1.45-14.3 pmol/L]). 68-Gallium DOTATATE positron emission tomography showed mild uptake in an anterior mediastinal nodule, and thymectomy revealed benign thymic tissue. Subsequent imaging identified a left upper lobe pulmonary nodule. Surgical resection confirmed a well-differentiated ACTH-producing carcinoid tumor. Excess cortisol likely contributed to CSCR through increased choroidal vascular permeability and subretinal fluid accumulation [1]. Prior studies suggest that a subset of patients with endogenous Cushing syndrome may develop CSCR [2]. This case highlights that CSCR can be an early manifestation of ectopic ACTH-dependent hypercortisolism and should prompt evaluation.

## Abbreviations

ACTH: adrenocorticotropic hormone
CSCR: central serous chorioretinopathy
